# Obstructive sleep apnea-hypopnea syndrome induced by dexmedetomidine in a patient with long-term sedatives: A case report

**DOI:** 10.1097/MD.0000000000048918

**Published:** 2026-06-05

**Authors:** Qin Feng, Hong Yin

**Affiliations:** a Department of Anesthesiology, Chengdu Fifth People’s Hospital (The Second Clinical Medical College, Affiliated Fifth People’s Hospital of Chengdu University of Traditional Chinese Medicine), Chengdu, China.

**Keywords:** dexmedetomidine, OSAHS, respiratory depression, sedative-hypnotic drugs

## Abstract

**Rationale:**

Dexmedetomidine is extensively recognized as a sedative featuring a relatively mild respiratory depressant effect. Nevertheless, for patients who are on long-term sedative use and potentially suffer from co-morbid insomnia and sleep apnea (COMISA), the safety of this drug remains ambiguous. This case report uncovers a rare yet severe adverse reaction associated with this drug.

**Patient concerns:**

A 62-year-old male with a 2-year history of insomnia has been on long-term use of multiple sedative agents. He reported persistent symptoms including poor sleep quality, daytime fatigue, and cognitive decline, along with a strong desire to reduce his medication burden.

**Diagnoses:**

During the patient’s hospitalization, polysomnography results confirmed that the patient suffered from severe obstructive sleep apnea-hypopnea syndrome (OSAHS). This condition was induced by the administration of dexmedetomidine during the infusion process.

**Interventions:**

Before conducting the sleep monitoring, CBTI (cognitive behavioral therapy for insomnia), stellate ganglion block, ozone enema, and traditional Chinese medicine treatment were performed. After the diagnosis of severe OSAHS was confirmed, dexmedetomidine was immediately discontinued. Subsequently, noninvasive ventilation was adopted as the main auxiliary treatment method.

**Outcomes:**

After the intervention measures were implemented, the patient’s sleep quality and oxygen supply condition significantly improved. During the follow-up, he successfully reduced his reliance on sedative-hypnotic drugs.

**Lessons:**

This case indicates that for patients with a long history of using sedatives, dexmedetomidine may cause or exacerbate severe OSAHS. Clinicians should be vigilant and consider conducting sleep apnea screening for high-risk patients before using sedatives and be prepared with noninvasive ventilation support equipment.

## 1. Introduction

Obstructive sleep apnea-hypopnea syndrome (OSAHS) is a common disorder characterized by recurrent episodes of complete or partial collapse of the pharyngeal airway during sleep. This leads to cessation or reduction of airflow (apnea or hypopnea), resulting in decreased oxygen saturation and fragmented sleep.^[1]^ Long-term use of sleeping pills (such as benzodiazepines) may pose a risk of worsening the symptoms of OSAHS.^[2]^ Benzodiazepines can increase the risk of acute respiratory failure in patients with OSAHS, and at the same time, they can cause damage to the body’s important defense mechanisms against hypoxemia and apnea.^[3]^ Dexmedetomidine is a highly selective α 2-adrenoceptor agonist. Dexmedetomidine is widely used in sedation treatment due to its advantages such as a low incidence of respiratory depression and a long duration of action.^[4]^ However, the drawbacks are that the onset of action is slow, the failure rate of sedation is high, environmental sensitivity is significant, and the half-life is long. There is still controversy regarding its impact on respiratory function. At our institution, we encountered a case where a patient with long-term sedative use developed severe OSAHS after dexmedetomidine administration, highlighting a potential risk that warrants clinical vigilance.

## 2. Case presentation

### 2.1. Patient information

A 62-year-old male, Mr. Deng (pseudonym), presented to the Sleep Medicine Center of Chengdu Fifth People’s Hospital seeking to improve his sleep and reduce his reliance on sleeping pills. Since 2021, he had experienced sleep disorders and received treatments at multiple hospitals, involving at least 3 different types of sleeping pills along with psychological treatment, with unsatisfactory results.

### 2.2. Chief complaint

The patient reported 2 years of difficulty initiating and maintaining sleep without any obvious cause. He woke feeling extremely fatigued after completely sleepless nights. His mood was significantly fluctuating and irritable. Previously diagnosed with “insomnia and anxiety state,” he was treated with Venlafaxine and Quetiapine, without significant improvement. His average sleep duration was about 4 hours per night. He complained of poor sleep quality, daytime fatigue, memory decline, dizziness, agitation, and a strong desire to reduce or discontinue sleeping pills.

### 2.3. Physical examination

This patient was conscious, oriented, and alert. His weight was 78.5 kg, height 167 cm, and body mass index 28.15 kg/m^2^. He was calm and cooperative but appeared in poor spirits. His voice pitch was high, and his mood was slightly irritable and anxious. His thought process was clear, logical, focused, and goal-oriented, with no perceptual disorder. He was afebrile, with normal blood pressure, stable vital signs, intact reflexes, and normal cranial nerves.

### 2.4. Previous medical history

The patient had visited the outpatient departments of West China Hospital and Sichuan Provincial People’s Hospital on multiple occasions due to sleep and emotional problems. None of the symptoms had been relieved, and the insomnia condition had not improved significantly. Therefore, the patient was hospitalized at the Department of Psychosomatic Medicine of Chengdu Fifth People’s Hospital in August 2023 due to “poor sleep and palpitations for 1 year, recurrence and aggravation for half a month.” After admission, no obvious abnormalities were found in the physical examination of heart, lungs, abdomen and nervous system. Specialized examination: The patient was admitted actively, dressed neatly and appropriately, with an anxious expression, able to take care of herself, cooperative, friendly to medical staff and family members, with concentrated attention, answering questions accurately, a low voice, good orientation, clear consciousness, and self-awareness. Depression and anxiety syndrome were elicited, but no psychotic symptoms such as hallucinations and delusions were elicited. Suicidal ideation was elicited, but no suicidal behavior was elicited. Social function was normal. Sleep monitoring was completed, and the diagnosis was mild obstructive sleep apnea syndrome. In terms of treatment, escitalopram oxalate sustained-release tablets were used to improve mood, quetiapine fumarate was used to enhance efficacy, clonazepam was used for sedation, and estazolam tablets were used to improve sleep. At the same time, CBTI psychological therapy, electroencephalogram therapy, and repetitive transcranial magnetic stimulation therapy were used as physical treatments. After the sleep condition improved, the drugs were changed to zopiclone and trazodone. Specifically, the patient had been on a long-term regimen of zopiclone (7.5 mg nightly) and trazodone (50 mg nightly), in addition to previously documented use of clonazepam and estazolam, reflecting a significant history of multi-drug sedative-hypnotic use.

### 2.5. Characteristics of the patient’s insomnia

The patient’s insomnia was characterized by long-term dependence on multiple sedative-hypnotics, typically involving 3 or more concurrent medications. The patient reported that since the previous treatment, he had been taking at least 3 or more sleep medications. His sleep pattern is to go to bed at 9:30 pm and fall asleep in about 30 minutes. He wakes up at 2 am and has no more sleepiness. He gets up at 6 am His sleep duration is merely 4 hours, which leads to daytime fatigue, a significant decline in memory, dizziness, and emotional agitation.

### 2.6. Management

#### 2.6.1. *Treatment measures received at the sleep center*:

Since admission to the hospital, the patient has received daily stellate ganglion block to regulate neurological functions, daily CBTI to improve sleep behavior cognition, daily rectal ozone perfusion to combat inflammation, daily repetitive transcranial magnetic stimulation to improve cerebral circulation, traditional Chinese medicine and cheek treatment for symptomatic adjustment, and dexmedetomidine for anti-anxiety treatment. During the period of intravenous infusion of dexmedetomidine, the patient experienced frequent awakenings during the night due to sleep disturbances. Therefore, dexmedetomidine was discontinued, and noninvasive ventilator treatment was adopted instead. After using the noninvasive ventilator, the patient was able to sleep soundly.

#### 2.6.2. Hospital course and outcome:

The patient was admitted to Chengdu Fifth People’s Hospital for treatment on November 11, 2024 and discharged on November 16, 2024. During this period, we conducted laboratory tests, imaging studies, sleep studies, and specific consultations to accurately assess her condition, as follows.

##### 2.6.2.1. Imaging modalities:

- Carotid and vertebral artery color Doppler ultrasound (2024-11-11): No obvious abnormalities were found in the bilateral carotid and vertebral arteries.
- Chest CT scan: Findings included emphysema, a few scattered chronic inflammatory changes in both lungs, a few bone spur-related pneumonia in the right lower lobe, a few small nodules in both upper lobes (stable, follow-up recommended), calcification in the left lower lobe, a few slightly enlarged mediastinal lymph nodes, a slightly enlarged heart shadow, calcification in the aortic and mitral valve areas, a slightly thickened pulmonary artery trunk, progressive degeneration of the chest wall, an old fracture of the left posterior rib, scattered low-density foci in the liver, fatty degeneration and atrophy of the pancreas, and a cystic lesion in the left kidney.
- Routine electrocardiogram examination (12-channel) (2024-11-11): Sinus rhythm, normal electrocardiogram.

##### 2.6.2.2. Laboratory results:

Upon admission and during the hospital stay, the patient had high transaminase and lipoprotein levels, while other laboratory results were generally normal.

##### 2.6.2.3. Sleep study:

Overnight polysomnography was performed and revealed severe OSAHS. The key quantitative parameters are summarized in Table 1.

**Table 1:** Polysomnography parameters.

Parameter	Finding
AHI	11.4/h
Obstructive apnea events	32 (longest: 236.0 s)
Hypopnea events	44 (longest: 56.0 s)
ODI	9.0/h
Mean oxygen saturation (SpO_2_)	94%
Nadir oxygen saturation (SpO_2_)	89%
Interpretation	OSAHS

AHI = apnea-hypopnea index, ODI = oxygen desaturation index, OSAHS = obstructive sleep apnea-hypopnea syndrome, PSG = polysomnography.

#### 2.6.3. Discharge and patient perspective:

After comprehensive treatment and noninvasive ventilator therapy, the patient’s sleep quality improved, the dosage of sleeping pills was reduced, and his overall condition improved by discharge. In his own words during follow-up, the patient reported feeling “much more rested during the day” and expressed relief at being able to “significantly reduce, and sometimes even completely stop, the sleeping pills,” which he had long desired. He noted his physical symptoms had “improved a lot” and his mental state was “greatly improved compared to before.” Subsequent telephone follow-up confirmed sustained reduction in sleeping pill use, with occasional complete cessation, alongside notable improvements in physical symptoms and mental well-being. To present the patient’s clinical course more clearly, Table 2 summarizes the timeline of key events, treatment measures and outcomes from the onset of symptoms to the follow-up period.

**Table 2:** Timeline of key clinical events, treatments, and outcomes.

Time point	Event/treatment	Outcome/status
2021	Onset of sleep disorders	Difficulty initiating/maintaining sleep
2021–2023	Multiple hospital treatments; Use of ≥ 3 sedatives; Psychological therapy	Poor sleep quality; Insomnia persisted
August 2023	Hospitalization in Psychosomatic Dept.; PSG: Mild OSAHS	Initiated pharmacotherapy (escitalopram, quetiapine, clonazepam, estazolam) & physical therapies (CBTI, rTMS); Sleep improved temporarily
Post-August 2023	Maintenance on zopiclone and trazodone	Sleep problems recurred/aggravated
November 11, 2024	Admission to sleep center	Comprehensive evaluation initiated
Hospital stay	Dexmedetomidine infusion for anxiety	Frequent nighttime awakenings; Suspected respiratory depression
Hospital stay	Discontinuation of dexmedetomidine; Initiation of noninvasive ventilation	Sleep quality improved significantly
November 16, 2024	Discharge	Improved sleep quality; reduced sedative requirement
Follow-up	Continued noninvasive ventilation; Reduced sedative use	Significant reduction/occasional cessation of sleeping pills; Improved physical and mental state

CBTI = cognitive behavioral therapy for insomnia, OSAHS = obstructive sleep apnea-hypopnea syndrome, PSG = polysomnography, rTMS = repeated transcranial magnetic stimulation.

## 3. Discussion

The uniqueness of this case stems from the observation that dexmedetomidine, a highly selective α-2 receptor agonist, has generally been considered to have a minor impact on respiratory function in previous studies.^[5]^ However, in this case, it turned out to be a direct trigger for severe OSAHS. This phenomenon may be related to the following unique background. One of the most likely factors is the cumulative effect of sedative drugs: patients who have been using benzodiazepines and non-benzodiazepines for a long time have their central respiratory drive suppressed and upper airway muscles relaxed, thereby increasing the potential risk of airway collapse.^[6]^ Dexmedetomidine does not directly inhibit the respiratory center, but its sedative effect may further aggravate airway obstruction through a synergistic effect. Then we analyzed the high-risk factors related to the patient’s anatomy and metabolism: the patient’s obesity (BMI 28.15) and abnormal structures of the nasopharynx (nasal cavity stenosis and hypertrophy of the turbinates) significantly increased the susceptibility to OSAHS. The sedative effect of dexmedetomidine may reduce the tension of the pharyngeal muscles,^[7]^ leading to a rapid deterioration of anatomical obstruction under the action of the drug. Finally, we concluded that the COMISA of the patients here was not fully identified: at the time of their initial diagnosis, the patients were only diagnosed with mild OSAHS, but no standardized intervention was carried out. Long-term sedative drugs masked the symptoms of OSAHS, forming a vicious cycle of “insomnia - drug dependence - airway deterioration.” The use of dexmedetomidine exposed the potential risks and suggested that COMISA patients should be prioritized for polysomnography (PSG) and airway assessment. Insomnia and obstructive sleep apnea were previously regarded as completely independent diseases.^[8]^ However, nowadays people are increasingly aware that insomnia and sleep apnea often occur simultaneously.^[9]^ Although the main clinical manifestations of each sleep disorder are significantly different, these 2 sleep disorders share many common symptoms, which may impede diagnosis and treatment. Therefore, management poses a challenge. Patients with undiagnosed OSAHS and insomnia can receive treatment for insomnia with hypnotic drugs and may continue such treatment for many years without improvement. However, once OSAHS is identified and properly treated, sleep problems may significantly improve.^[10]^ This case also provides us with many clinical insights.

Firstly, dexmedetomidine may pose unforeseen respiratory risks in patients with long-term dependence on sedative drugs, obesity, or with anatomical abnormalities of the airways. Therefore, strict monitoring of oxygenation and ventilation function is necessary.

Secondly, the treatment of COMISA patients should follow the principle of “correcting OSAHS first, then managing insomnia,” avoiding the indiscriminate use of sedative drugs. Noninvasive ventilation such as continuous positive airway pressure can be used as a first-line intervention method,^[11]^ which can not only improve oxygenation but also reduce sedative drug dependence.

Furthermore, the patient’s long-term use of GABAergic sedatives (zopiclone, trazodone, and previously benzodiazepines) likely established a vulnerable physiological substrate. Chronic exposure to such agents can lead to pharmacological tolerance and altered receptor sensitivity, potentially resulting in blunted central chemoreflex responses to hypercapnia/hypoxia and impaired neuromuscular compensatory mechanisms that maintain upper airway patency during sleep.^[12,13]^ When dexmedetomidine—a potent α_2_-adrenergic agonist with its own distinct suppressive effects—was introduced, it likely acted synergistically on this already compromised respiratory control system, precipitating the acute manifestation of severe OSAHS.

Finally, this case is a rare report of OSAHS induced by dexmedetomidine, suggesting that clinicians need to reexamine the safety of this drug in specific populations and promote the formulation of individualized treatment guidelines for COMISA.^[13]^ Screening for OSAHS before sedative use in chronic insomnia patients and considering the risks of polypharmacy in long-term insomnia management are crucial takeaways.

Clinical Implications and Lessons: This case offers several critical takeaways for clinical practice:

1. Pre-sedation Screening: A thorough history of sedative/hypnotic use and symptoms suggestive of sleep-disordered breathing should be actively sought before procedures requiring sedation. In high-risk patients, formal preoperative sleep apnea screening should be considered.^[14]^
2. Vigilant Monitoring: The notion of dexmedetomidine’s “respiratory-sparing” effect should be applied with caution in sedative-tolerant patients. Continuous respiratory monitoring (including pulse oximetry and ideally capnography) is imperative during and after its infusion in this population.
3. Individualized Treatment Plans: For patients with known or suspected COMISA, management should prioritize treating OSAHS (e.g., with continuous positive airway pressure) before addressing insomnia, avoiding the indiscriminate use of sedative-hypnotics that can exacerbate respiratory events.

## 4. Conclusion

This case highlights the potential risk of dexmedetomidine inducing or aggravating OSAHS in patients with a history of long-term sleeping pill use. It reveals how long-term, high-dose sedative use can exacerbate underlying airway obstruction in insomnia patients. This warns clinicians to thoroughly assess sleep and respiratory status and intensify respiratory monitoring to prevent serious complications in this vulnerable population.

## Acknowledgments

We sincerely express our gratitude to all the anesthesiologists, clinical doctors and nurses who have participated in providing care and conducting professional management for patients in our sleep disorder center. We have confirmed that no individuals are named without their permission.

## Author contributions

**Conceptualization:** Qin Feng, Hong Yin.

**Data curation:** Qin Feng.

**Formal analysis:** Qin Feng.

**Investigation:** Qin Feng.

**Writing—original draft:** Qin Feng.

**Writing—review & editing:** Qin Feng, Hong Yin.

**Supervision:** Hong Yin.
